# Effects of Naringin on Cardiomyocytes From a Rodent Model of Type 2 Diabetes

**DOI:** 10.3389/fphar.2021.719268

**Published:** 2021-08-23

**Authors:** A. Uryash, A. Mijares, V. Flores, J. A. Adams, J. R. Lopez

**Affiliations:** ^1^ Department of Neonatology, Mount Sinai Medical Center, Miami, FL, United States; ^2^ Centro de Biofísica y Bioquímica, Instituto Venezolano de Investigaciones Científicas, Caracas, Venezuela; ^3^ Department of Research, Mount Sinai Medical Center, Miami, FL, United States

**Keywords:** diabetes, naringin, cardiomyopathy, calcium, KATP channels, ROS, nicorandil, glibenclamide

## Abstract

Diabetic cardiomyopathy (DCM) is a primary disease in diabetic patients characterized by diastolic dysfunction leading to heart failure and death. Unfortunately, even tight glycemic control has not been effective in its prevention. We have found aberrant diastolic Ca^2+^ concentrations ([Ca^2+^]_d_), decreased glucose transport, elevated production of reactive oxygen species (ROS), and increased calpain activity in cardiomyocytes from a murine model (db/db) of type 2 diabetes (T2D). Cardiomyocytes from these mice demonstrate significant cell injury, increased levels of tumor necrosis factor-alpha and interleukin-6 and expression of the transcription nuclear factor-κB (NF-κB). Furthermore, decreased cell viability, and reduced expression of Kir6.2, SUR1, and SUR2 subunits of the ATP-sensitive potassium (K_ATP_) channels. Treatment of T2D mice with the citrus fruit flavonoid naringin for 4 weeks protected cardiomyocytes by reducing diastolic Ca^2+^ overload, improving glucose transport, lowering reactive oxygen species production, and suppressed myocardial inflammation. In addition, naringin reduced calpain activity, decreased cardiac injury, increased cell viability, and restored the protein expression of Kir6.2, SUR1, and SUR2 subunits of the K_ATP_ channels. Administration of the K_ATP_ channel inhibitor glibenclamide caused a further increase in [Ca^2+^]_d_ in T2D cardiomyocytes and abolished the naringin effect on [Ca^2+^]_d_. Nicorandil, a K_ATP_ channel opener, and nitric oxide donor drug mimic the naringin effect on [Ca^2+^]_d_ in T2D cardiomyocyte; however, it aggravated the hyperglycemia in T2D mice. These data add new insights into the mechanisms underlying the beneficial effects of naringin in T2D cardiomyopathy, thus suggesting a novel approach to treating this cardiovascular complication.

## Introduction

Type 2 Diabetes mellitus (T2D) is public health threat with a significant increase in affected individuals worldwide in the last decade (Zimmet et al., 2016). Globally the associated healthcare cost of diabetes has been projected to exceed $2.1 trillion by 2030 (Bommer et al., 2018). Epidemiologic data show that the frequency of heart diseases in diabetic patients is much higher, and the prognosis is worse than in populations without diabetes (Thrainsdottir et al., 2005; Lehrke and Marx, 2017; Singh et al., 2018). One significant complication associated with type 2 diabetes is diabetic cardiomyopathy (DCM), characterized by diastolic dysfunction, ventricular hypertrophy, and interstitial fibrosis leading to heart failure in the absence of dyslipidemia, hypertension and coronary artery disease (Nicolino et al., 1995; Redfield et al., 2003; Singh et al., 2018; Dillmann, 2019). While hyperglycemia provokes noticeable effects on cardiomyocyte function, blood glucose levels are not necessarily predictive of cardiac dysfunction (Diamant et al., 2003). Management of T2D - DCM involves lifestyle modifications, including diet, regular physical activity, and pharmacological therapies for glucose management, lipid abnormalities, hypertension, and cardiac failure (Pappachan et al., 2013). Although hyperglycemia appears to drive the pathogenesis of DCM, firm glycemic control in diabetic patients has not translated into a significant reduction in either morbidity or mortality (Wang and Reusch, 2012). Unfortunately, despite decades of intense clinical research, no therapies have substantially protected DCM development in diabetic patients.

Increasing evidence supports the beneficial anti-inflammatory, hypoglycemic, and antioxidant effects of 4′,5,7-trihydroxyflavanone-7-rhamnoglucoside (naringin), a natural polyphenol bioflavonoid that is abundant in grapefruit and related citrus species (Rajadurai and Stanely Mainzen Prince, 2006; Sharma et al., 2011; Mahmoud et al., 2012; Alam et al., 2014). As a hypoglycemic, naringin increases hepatic glycolysis and glycogen concentration and decreases hepatic gluconeogenesis, blood glucose, and glycosylated hemoglobin levels (Jung et al., 2004; Jung et al., 2006). Furthermore, naringin has been shown to have cardioprotective properties against high glucose-induced injuries (Huang et al., 2013; Chen J. et al., 2014; You et al., 2016). However, the specific mechanisms underlying the cardioprotective effects of naringin are not fully understood.

The study aimed to investigate the potential cardioprotective effect of naringin in cardiomyocytes isolated from T2D mice. Our results demonstrate that the naringin exerts cardioprotection through multiple mechanisms 1) Reducing [Ca^2+^]_d_; 2) Improving cardiomyocyte glucose uptake; 3) Decreasing production ROS; 4) Reducing the level of myocardial tumor necrosis factor-α (TNF-α) and interleukin-6 (IL-6) and the expression of NF-κB; 5) Attenuating calpain activity, cardiac injury and improving cardiomyocyte viability; 6) Increasing the expression of Kir6.2, SUR1, and SUR2 subunits of the ATP-sensitive potassium (K_ATP_) channels. In addition, our results provide new insight into the mechanisms by which naringin exercises cardioprotection in T2D cardiomyocytes.

## Material and Methods

### Animals

Male homozygous C57BL/10 (WT-control) and C57BL/KsJ-db/db (T2D) mouse colonies were established using founders obtained from the Jackson Laboratory (Bar Harbor, ME, United States). Control and T2D 12 weeks old mice were housed under a constant temperature (24°C) and 12-h light/12-h dark cycle, with free access to water and fed with pelletized commercial chow diet. All experimental animal procedures were carried out following the Care and Use Handbook of Laboratory Animals published by the US National Institute of Health (NIH publication No. 85–23, revised 1996) and approved by the Institutional Animal Care and Use Committees at the Mount Sinai Medical Center, Miami Beach, FL, United States.

### Diabetic Model

12-week old C57BL/KsJ-db/db mice, which spontaneously develop hyperglycemia due to a leptin receptor mutation with phenotypic features of human T2D, were used as our mouse model of T2D (Hummel et al., 1966). We used fasting blood glucose levels to corroborate the diabetic condition. Mice with a 12 h fasting glucose level >250 mg/dl for three consecutive tests were considered diabetic and used experimentally.

### Mouse Blood Collection and Glucose Determinations

Blood samples (5 µL) for glucose measurements were obtained from 12 h fasting T2D and control mice from the tail vein on two occasions before beginning the treatment protocol and after the 4 weeks of treatment immediately prior the removing the heart. Blood glucose concentrations were measured on a glucometer (AlphaTRAK^®^ Glucose Meter, Abbott Animal Health, Abbott Park, IL United States) (Andrikopoulos et al., 2008).

### Pharmacological Treatments

#### Naringin Treatment

Control and T2D mice (12 weeks old) were treated with naringin 20, 40, or 60 mg/kg/day (d) dose (Sigma-Aldrich, MO United States) administered intraperitoneally (i.p.) once daily for 4 weeks. Untreated control and T2D mice received equal volumes of normal saline solution administered i. p. daily for the same period. Mice were divided randomly into eight groups. ***Group 1***. Control mice in which no therapeutic intervention was made during the study (n = 20 mice); ***Group 2***: Control mice treated with naringin (20 mg/kg/d i. p.) (n = 5 mice); ***Group 3:*** Control mice treated with naringin (40 mg/kg/d i. p.) (n = 5 mice); ***Group 4:*** Control mice treated with naringin (60 mg/kg/d i. p.) (n = 20 mice); ***Group 5***. T2D mice in which no therapeutic intervention was made during the study (n = 20 mice); ***Group 6***: T2D mice treated as ***Group 2,*** (n = 5 mice); ***Group 7***: T2D mice treated as ***Group 3,*** (n = 5 mice); ***Group 8***: T2D mice treated as ***Group 4*** (n = 20 mice)***.*** At the end of the 4 weeks treatment, all mice were fasted for 12 h, heparinized (1000 U/kg, i. p.), anesthetized with ketamine (100 mg/kg), and xylazine (5 mg/kg), and the heart was removed to prepare isolated cardiomyocytes. Blood samples were drawn from the tail vein for a final glucose determination before the heart was removed.

#### Glibenclamide Treatment

In the second cohort of mice (12 weeks old), we examined the importance of K_ATP_ channels on the naringin cardioprotective effects in T2D cardiomyocytes by testing the effect of the K_ATP_ channel blocker glibenclamide. For this study, mice were divided into six groups (n = 5 mice per group). Group 1. Control mice that did not receive therapeutic intervention; Group 2. Control mice treated with glibenclamide (10 μg/kg i. p./d) for 4 weeks; Group 3: Control mice treated with glibenclamide (10 μg/kg, i. p./d) for 48 h and then with glibenclamide (10 μg/kg, i. p.) in combination with naringin (60 mg/kg i. p./d) for 4 weeks; Group 4. T2D mice that did not receive therapeutic intervention; Group 5: T2D mice were treated as Group 2; Group 6: T2D mice were treated as Group 3. Untreated control and T2D mice received equal volumes of normal saline solution administered i. p. daily for the same period. After the 4 weeks of treatment, cardiomyocytes were isolated from all experimental animals, following the protocol previously described for naringin treatment above.

#### Nicorandil Treatment

In the third cohort of mice (12 weeks old), we assessed whether a K_ATP_ channel agonist could imitate the cardioprotective effect of naringin in T2D cardiomyocytes regarding the [Ca^2+^]_d_ and ROS production. Mice were divided into four groups (n = 5 mice per group). Group 1. Control mice in which no therapeutic intervention was made during the study; Group 2. Control mice treated orally with nicorandil (10 mg/kg/d) for 4 weeks; Group 3: T2D mice in which no therapeutic intervention was made during the study; Group 4: T2D mice were treated as Group 2. After the 4 weeks of treatment, cardiomyocytes were isolated from all experimental animals, following the protocol previously described for naringin treatment above. Blood samples were obtained for glucose determination before the heart was removed.

### Isolation of Cardiomyocytes and Inclusion Criteria

Hearts from anesthetized mice were rapidly removed, attached to a cannula, and mounted onto a temperature-controlled (37°C) Langendorff reverse coronary perfusion system for enzymatic dissociation of cardiomyocytes as previously described (Liao and Jain, 2007). All cardiomyocytes utilized in this study (within 5 h after isolation) were rod-shaped, had well-defined striation spacing, did not contract when perfused with Tyrode solution containing Ca^2+^, and had a resting sarcomere length of ≥1.75 µm (Wussling et al., 1987) after being electrically stimulated (1 ms square pulse, X1.5 threshold voltage, 0.5 Hz, for 3 min).

### Ca^2+^-Selective Microelectrodes

**FIGURE 1:**
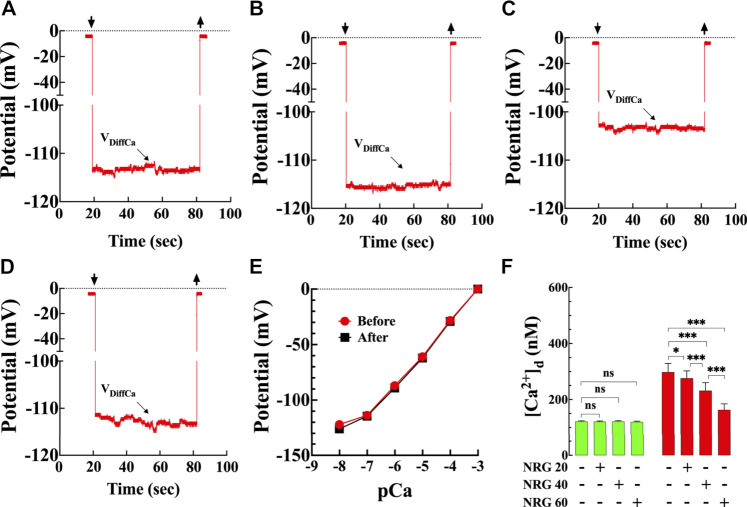
Effects of naringin on diastolic [Ca^2+^] in T2D cardiomyocytes. Representative traces of [Ca^2+^]_d_ recorded from enzymatically isolated mouse ventricular cardiomyocytes using double-barreled ion-selective microelectrodes. **(A)** control with no therapeutic intervention, [Ca^2+^]_d_ = 124 nM. Top left arrow: Ca^2+^ microelectrode cell impalement, and top right arrow: microelectrode cell withdrawing. **(B)** control treated with naringin (60 mg/kg i. p.), [Ca^2+^]_d_ = 119 nM. **(C)** T2D with no therapeutic intervention, [Ca^2+^]_d_ = 306 nM. **(D)** T2D treated with naringin (60 mg/kg i. p.), [Ca^2+^]_d_ = 170 nM. The V_DiffCa_ potential was converted to [Ca^2+^] using the corresponding microelectrode calibration curve. **(E)** Typical calibration curves of submicron tip Ca-sensitive microelectrodes before and after [Ca^2+^]_d_ determinations (Lopez et al., 1985; Eltit et al., 2013). The microelectrode shows a Nernstian slope (28.5 mV per pCa unit) (pCa = −log [Ca^2+^]) between pCa 3 and pCa 7 and still have a helpful response between pCa7 and 8. **(F)** Dose-response effects of naringin on [Ca^2+^]_d_ measured in quiescent cardiomyocytes isolated from control (green) and T2D mice (red). [Ca^2+^]_d_ was significantly higher in T2D than control cardiomyocytes (*p* < 0.001). Naringin reduced significantly [Ca^2+^]_d_ in a dose-dependent manner in T2D cardiomyocytes compared to untreated cardiomyocytes (*p* < 0.001), with no significant effect on control cardiomyocytes (*p* = 0.99 compared to untreated). Experiments were conducted in nmice = 5–20/group and ncells = 10–21/group. Values are expressed as means ± S.D. Statistical analysis was performed using one-way ANOVA, followed by Tukey’s multiple comparison tests, ns *p* > 0.05, *p < 0.05, ***p < 0.001.

The procedure for manufacturing the doubled-barreled Ca^2+^ selective microelectrodes has been described previously (Eltit et al., 2013). Ca^2+^ ionophore II (ETH 129) (Sigma-Aldrich, MO, United States) was used to backfill the microelectrode tip and pCa 7 (pCa = −log free [Ca^2+^]) the shanks of the Ca^2+^- selective barrel. 3M KCl was used to fill the tip and shank of the membrane potential barrel. Each ion-selective microelectrode was individually calibrated in solutions with known [Ca^2+^] (pCa3 to pCa8), and only those microelectrodes with Nernstian slope (28.5 mV per pCa) between pCa3 and 7 were used experimentally (Lopez et al., 1983; Lopez et al., 2000; Eltit et al., 2013) (Figure 1E). Ca^2+^-selective microelectrodes were calibrated again after completing [Ca^2+^]_d_ measurements, and if the two calibrations curves showed a difference greater than 3 mV (pCa6—7), the data was discarded.

### Recording of Diastolic Calcium Concentration

Isolated cardiomyocytes were impaled with double-barreled Ca^2+^-selective microelectrodes for the determination [Ca^2+^]_d_, and the potentials were recorded *via* a high-impedance amplifier (WPI 773 electrometer, FL, United States) as described previously (Lopez et al., 2000; Lopez et al., 2017). The potential from the 3M KCl microelectrode barrel (V_m_) was subtracted electronically from the potential recorded by the Ca^2+^ ion-selective barrel (V_CaE_) to produce a differential Ca^2+^-specific potential (V_DiffCa_), which represent the cardiomyocyte [Ca^2+^]_d_. V_m_ and V_DiffCa_ potentials were acquired at a frequency of 1,000 Hz with AxoGraph software (version 4.6; Axon Instruments, CA, United States) and stored in a computer for further analysis. The following criteria were used to accept individual cardiomyocytes [Ca^2+^]_d_ measurements from control and db/db mice: 1) a quick drop to a steady level of Vm; 2) a stable recording potential (V_m_ and V_DiffCa_) for no less than 60 s; 3) a quick return to baseline of Vm and V_DiffCa_ potential on the exit of the microelectrodes from the cell. These criteria were not met in 15% of the total impalements carried out in control and 25% in db/db cardiomyocytes, and these data were rejected from our analysis.

### Cardiomyocyte Glucose Uptake

Glucose uptake was assessed by measuring the change in fluorescence of 2-(N-(7-Nitrobenz-2-oxa-1,3-diazol-4-yl) Amino)-2-Deoxyglucose (2-NBDG; Invitrogen, CA, United States) in isolated cardiomyocytes (Gallo et al., 2018). Enzymatically isolated cardiomyocytes were 2-NBDG (250 µM) loaded for 30 min in the dark in glucose-free Tyrode solution. After the loading was completed, cardiomyocytes were washed with Tyrode standard solutions and then imaged using an invert fluorescence microscope (Zeiss Axiovert 200, NY, United States), equipped with a 50X/0.90 oil-immersion objective (Zeiss, NY, United States). Excitation light and emission light wavelengths were set at 475 and 535 nm, respectively, utilizing a filter set (XF100–2, Omega Optical, VT, United States). At the end of the experiment, only cardiomyocytes contracted in response to electrical stimulation (1 ms square pulse, X1.5 threshold voltage, at 37°C) were included. Glucose uptake for all groups was recorded for 3 min and normalized to the value from untreated control cardiomyocytes.

### Measurement of Reactive Oxygen Species (ROS)

Intracellular ROS production was measured in isolated cardiomyocytes using the cell-permeant probe, 2′, 7′- dichlorodihydrofluorescein diacetate (DCFDA) (Sigma-Aldrich, MO, United States). Cardiomyocytes were incubated with 5 µM DCFDA for 30 min (37°C) (Chang et al., 2019) followed by 15 min washout with Tyrode standard solution. The uncharged nonfluorescent DCFDA enters the cell by passive diffusion, where it is hydrolyzed to DCFH by intracellular esterase’s. In the presence of ROS, DCFH is oxidized to the highly fluorescent dichlorofluorescein (DCF). Loaded cardiomyocytes were transferred to an experimental chamber and mounted on the stage of an inverted fluorescence microscope (Zeiss Axiovert 200, NY, United States). DCF fluorescence was measured at 480 and 535 nm as excitation and emission wavelengths and recorded for 60–70 s at 37°. Only cardiomyocytes contracted in response to electrical stimulation (1 ms square pulse, X1.5 threshold voltage, at 37°C) at the end of the experiment were included. The rate of DCF fluorescence for all groups was normalized to the value from untreated control cardiomyocytes.

### Calpain Activity Assay

Calpain activity was assessed in isolated cardiomyocytes using a fluorometric calpain activity assay kit (Abcam Inc, MA, United States). The lysates of cardiomyocytes were centrifuged, and the supernatant was used to detect calpain activity using the fluorescent substrate N-succinyl-LLVY-AMC. Fluorescence was detected using a microplate reader (Molecular Device, CA, United States) at 360 nm excitation and 460 nm emission wavelengths. Fluorescence measured in arbitrary units for all groups was normalized to values from untreated control cardiomyocytes.

### Assay for Cardiac Injury and Cell Viability

Lactate dehydrogenase (LDH) release, a marker of cellular injury, was measured in the medium in naringin-treated and untreated control and T2D cardiomyocytes using an LDH assay kit (Sigma-Aldrich, MO, United States) according to the manufacturer’s instructions. The activity of LDH was normalized to values from untreated control cardiomyocytes.

Cardiomyocyte viability was assessed by 3-(4,5-dimethylthiazol-2-yl)-2,5-diphenyltetrazolium bromide (MTT) assay following the manufacturer’s instructions (Abcam Inc, MA, United States). Data are expressed as a reduction in MTT concentration compared to untreated control cardiomyocytes.

### Measurement of TNF-α and Interleukin-6 Concentrations and Western Blot Analyses

A different cohort of naringin-treated (60 mg/kg/d i. p. for 4 weeks) and untreated control and T2D mice (12 weeks old) were divided randomly into four groups (n = 5 mice per group) for the determination of TNF-α and IL-6. Mice were anesthetized, the heart was removed and homogenized in 10% phosphate buffer to measure the inflammatory mediators TNF-α and IL-6 using ELISA kits (Abcam Inc, MA, United States). The measurements of these markers were carried out according to the manufacturer’s protocols using a microplate reader (Molecular Devices, CA, United States).

For Western blot analysis, naringin-treated (60 mg/kg/d i. p. for 4 weeks) and untreated control and T2D mice (12 weeks old) (nmice = 3 per group) mice were anesthetized; the heart was removed, the ventricle dissected and then homogenized in radioimmunoprecipitation assay (RIPA) buffer for protein extraction. After protein transfer from the gel to the membrane, we sliced the membrane horizontally according to the protein molecular weight of interest (Uryash et al., 2015). The protein size was determined based on the protein standard marker. Separate membrane strips were incubated with the primary KATP antibody (Kir6.2, SUR1, SUR2, Abcam Inc, MA, United States) and NF-κB (Abcam Inc, MA, United States) 1:2,500 dilution. The intensity of the bands of these proteins was normalized to the intensity of the housekeeping protein ß-actin, which was used as a loading control (Actin, Abcam Inc, MA, United States).

### Solutions

All solutions were made using ultrapure water supplied by a Milli-Q system (Millipore, Bedford, MA). Tyrode solution had the following composition (in mM): NaCl 130, KCl 2.68, CaCl_2_ 1.8, MgCl_2_ 1, glucose 5, NaHCO_3_ 20, NaH_2_PO 0.9, gassed with 95% O_2_ and 5% CO_2_, pH 7.2. For the conditions where a Tyrode glucose-free solution was required, glucose was omitted from the solution. All experiments were performed at 37°C.

### Statistical Analysis

Experimental results are expressed as means ± SD; *n*_*mice*_ represents the number of mice used and *n*_*cells*_ the number of successful measurements used for statistical analysis. Data were analyzed using one-way ANOVA for repeated measures, followed by Tukey’s multiple comparison tests to determine significance. For Western blots, statistical analyses were carried out using the paired Student’s independent *t*-test. A *p*-value of less than 0.05 was considered significant. GraphPad Prism 9 (GraphPad Software, CA, United States) was used for statistical analysis.

## Results

### Naringin Reduces the Elevated [Ca^2+^]_d_ in T2D Cardiomyocytes in Dose-Dependent

Representative records of naringin effect on [Ca^2+^]_d_ measured in control and T2D cardiomyocytes (Figures 1A–D) using calcium-selective microelectrodes (Figure 1E). In control cardiomyocytes, the average [Ca^2+^]_d_ was 122 ± 2 nM compared to 298 ± 31 nM in T2D cardiomyocytes (*p* < 0.001) (Figure 1F). Naringin reduced [Ca^2+^]_d_ in T2D cardiomyocytes in a dose-dependent manner (Figure 1F). In T2D cardiomyocytes 20 mg/kg/d reduced [Ca^2+^]_d_ to 276 ± 26 nM (7%), 40 mg/kg/d to 232 ± 29 nM (22%) and 60 mg/kg/d to 163 ± 21 nM (45%) (Figure 1F) (*p* < 0.001 compared to untreated T2D cardiomyocytes) but had no effect on [Ca^2+^]_d_ in control at any of the tested doses (*p* > 0.05) (Figure 1F). Because naringin 60 mg/kg/d dose had the optimum effect on lowering [Ca^2+^]_d_ in T2D cardiomyocytes, it was adopted as the standard dose for all other experiments described in this study.

### Naringin Enhanced Cardiomyocyte Glucose Transport and Reduced Hyperglycemia

**FIGURE 2:**
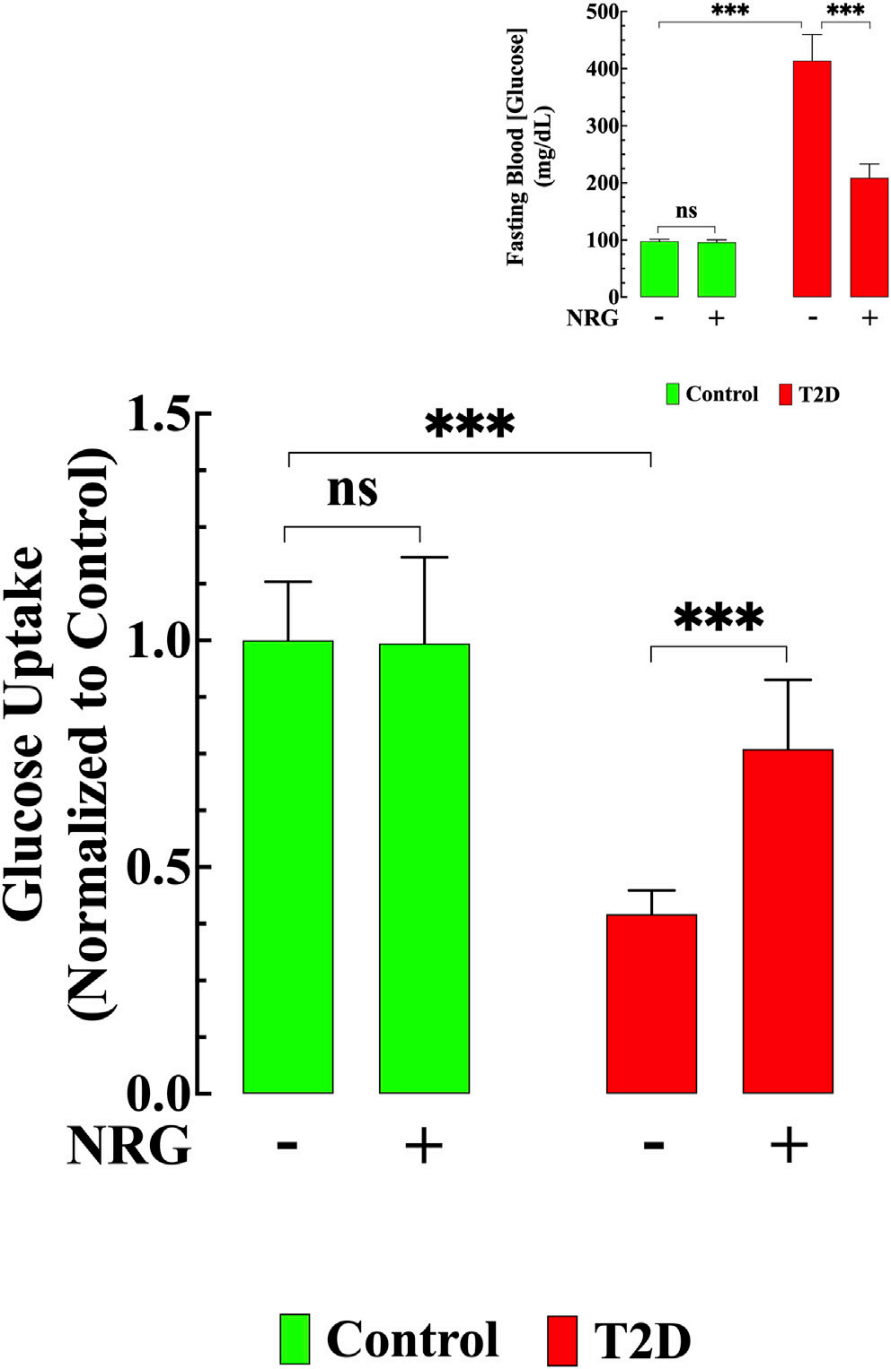
Naringin improved glucose transport and lowered hyperglycemia. Basal glucose uptake in T2D cardiomyocytes was significantly reduced compared to control (*p* < 0.001). Naringin treatment provoked a significant increase in glucose uptake in T2D cardiomyocytes compared to untreated T2D cardiomyocytes (*p* < 0.001). Naringin treatment did not modify cardiomyocytes’ glucose uptake in control cardiomyocytes (*p* = 0.99). Glucose uptake experiments were conducted in *n*_*mice*_ *=* 5*/*group; *n*_*cells*_ *=* 19–21/group. Insert shows the significant hypoglycemic effect of naringin in T2D mice (*p* < 0.001), however, no effect was observed in naringin-treated control mice (*p* = 0.36 compared to untreated mice). Blood Glucose was measured in *n*_*mice*_ *=* 20/group; *n*_*determinations*_ *=* 15–20/group before and after naringin treatment. Values were normalized to control untreated cardiomyocytes (glucose uptake) or indicated as mg/dL (blood glucose) and expressed as means ± S.D. Statistical analysis was performed as described before, ns *p* > 0.05, ***p < 0.001.

The T2D heart is characterized by a decrease in glucose uptake (Jaswal et al., 2011). To further elucidate the effect of naringin, we carried out glucose uptake measurements in cardiomyocytes isolated from naringin-treated and untreated control and T2D mice using the fluorescent glucose analog 2-NBDG. Basal glucose uptake was reduced (60%, *p* < 0.001) in T2D cardiomyocytes compared to controls (Figure 2). Naringin treatment (60 mg/kg/d) induced a significant increase in glucose uptake in T2D cardiomyocytes (111%, compared to untreated T2D cardiomyocytes, *p* < 0.001). There was no significant difference in the control group between untreated and naringin-treated cardiomyocytes (*p* = 0.99) (Figure 2).

The average blood glucose levels in fasting untreated T2D mice was 414 ± 45 mg/dl compared to 98 ± 4 mg/dl in untreated control mice (*p* < 0.001) (Insert Figure 2). Naringin treatment (60 mg/kg/d) for 4 weeks significantly reduced blood glucose in the T2D group (209 ± 24 mg/dl, *p* < 0.001), but it had no significant effect in controls (96 ± 4 mg/dl*, p =* 0.21) (Insert Figure 2).

### Attenuation of Diabetes-Associated Oxidative Stress by Naringin

**FIGURE 3:**
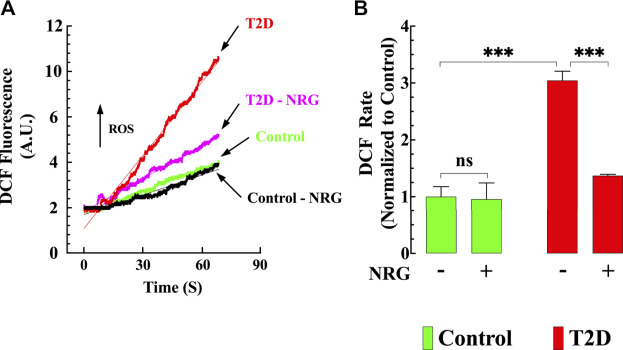
Naringin reduced intracellular ROS generation T2D cardiomyocytes. DCF fluorescence signal rate was significantly enhanced in T2D compared to control cardiomyocytes. **(A)** Representative records of DCF fluorescence signal from control (black), control naringin treated (green), T2D (red), and T2D naringin treated (purple) cardiomyocytes. **(B)** Average rate of DCF fluorescence signal from all cardiomyocytes groups. DCF fluorescence signal measurements were obtained from *n*_*mice*_ *= 5/*group; *n*_*cells*_ *=* 20–27/group. Values were normalized to control untreated cardiomyocytes and expressed as means ± S.D. Statistical analysis was performed as described before, ns *p* > 0.05, ***p < 0.001.

Oxidative stress plays a prominent role in the pathogenesis of diabetes (Guo et al., 2018). Therefore, cardiomyocyte oxidant levels for all groups were quantified using DCFDA fluorescence probe. Figure 3A shows representative records of the rate of dichlorofluorescein (DCF) fluorescence obtained from naringin treated and untreated control and T2D cardiomyocytes. Cardiomyocytes from T2D mice had a significantly increased DCF fluorescence signal rate compared to controls (204%) (*p* < 0.001) (Figure 3B). Naringin treatment (60 mg/kg/d) significantly reduced the rate of DCF fluorescence in T2D cardiomyocytes by 55% (*p* < 0.001 compared to untreated T2D) but did not affect naringin-treated control cardiomyocytes (*p* = 0.91 compared to the untreated control group) (Figures 3A,B).

### Naringin Suppressed Diabetes-Induced Myocardial Inflammation

**FIGURE 4:**
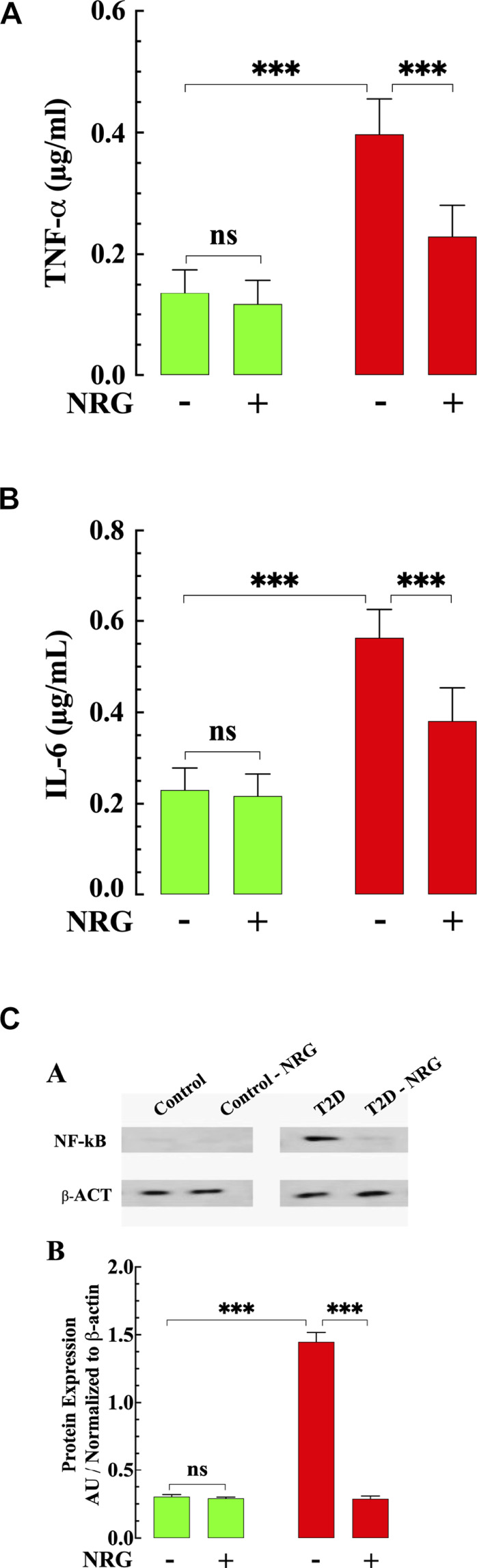
Effects of naringin on TNF-α, IL-6, and NF-kB in T2D ventricular tissues. **(A)** T2D ventricular tissue had a significantly TNF-α higher concentration than control ventricular cell (*p <* 0.001). Naringin treatment significantly reduced TNF-α levels in T2D tissues compared to untreated T2D (*p <* 0.001). Naringin treatment did not affect TNF-a levels in control ventricular tissue (*p =* 0.98 compared to untreated control). **(B)** Ventricular IL-6 levels were significantly increased in T2D heart tissues than untreated control tissues (*p <* 0.001). Naringin significantly lowered tissue IL-6 in T2D ventricular cells (*p <* 0.001 compared to untreated ventricular cells). No effect on basal IL-6 levels was observed in control naringin-treated ventricular tissue compared to untreated control (*p =* 0.30). **(C) Top:** representative Western blotting analysis of NF-kB in myocardium from naringin-treated and untreated-control and T2D mice. **Bottom**: Densitometric analysis of Western blot experiments. Expression of p65- NF-kB was significantly upregulated in T2D myocardium tissue compared to control tissue (*p* < 0.001). Naringin treatment normalized the expression p65- NF-kB in T2D cardiac tissue (*p* < 0.001) with no effect on control (*p* = 0.97). Data were normalized to housekeeping protein ß-actin and expressed. TNF-α and IL-6 measurements were obtained from *n*_*mice*_ *=* 5 mice/group, *n*_*determinations*_ = 10/group. Western blot experiments were carried out in *n*_*mice*_ = 3 mice/group, *n*_*WB*_ = 4/group. Values are expressed as means ± S.D. Statistical analysis was performed as described before, ns *p* > 0.05, ***p < 0.001.

Previous results have shown that an increase of inflammatory cytokines plays a critical role in developing diabetic cardiomyopathy (Palomer et al., 2013). We examined the effect of naringin on the levels of TNF-α and IL-6 in mice myocardium from naringin-treated and untreated-control and T2D mice. The content of proinflammatory cytokines TNF-α and IL-6 were significantly higher in myocardial tissue from T2D mice compared to control (191 and 145% respectively) (*p* < 0.001 compared to control, Figures 4A,B). Treatment with naringin (60 mg/kg/d) significantly reduced myocardial TNF-α and IL-6 levels in T2D by 42 and 32%, respectively (*p* < 0.001 compared to untreated T2D mice (Figures 4A,B). Naringin treatment had no significant effect on TNF-α and IL-6 control myocardium (*p* = 0.98 TNF-α and p = 0.30 for IL-6).

Since NF-κB is an important mediator in the pathophysiology of DCM (Lorenzo et al., 2011), we studied the expression of NF-κB protein in myocardium from naringin-treated and untreated-control and T2D mice. NF-κB expression was significantly upregulated in T2D tissue (360%) compared with control (*p* < 0.001) (Figure 4C). Treatment with naringin (60 mg/kg/d) normalized the expression of NF-κB in T2D myocardium compared to untreated tissue (*p* < 0.001) (Figure 4C), without affecting the expression in control naringin treated myocardium (*p =* 0.94).

### Reduction of Calpain Activity in T2D Cardiomyocytes by Naringin

**FIGURE 5:**
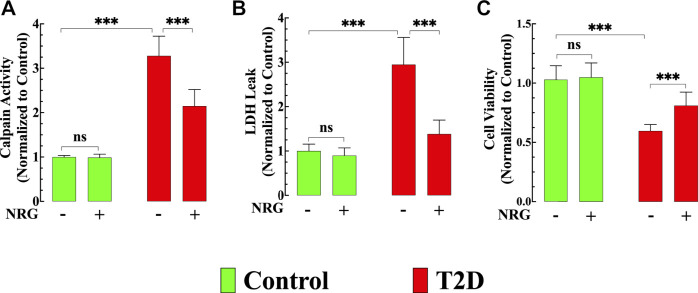
Effects of naringin on calpain activity, cardiac injury, and cell viability. **(A)** Calpain activity was significantly increased in T2D cardiomyocytes compared to control cardiomyocytes (*p* < 0.001). Naringin treatment significantly reduced calpain activity compared to untreated T2D cardiomyocytes (*p* < 0.001). Naringin treatment did not affect basal calpain activity in control cardiomyocytes (*p* = 0.99 compared to untreated). Calpain data was obtained from *n*_*mice*_ *=* 5/group; *n*_*cells*_ *=* 15–20/group. **(B)** LDH leak was significantly higher in T2D cardiomyocytes compared to control cardiomyocytes (*p* < 0.001). Naringin treatment significantly diminished the LDH leak compared to untreated T2D cardiomyocytes. Naringin did not modify basal LDH leak in control cardiomyocytes (*p =* 0.81 compared to untreated). LDH measurements were obtained from *n*_*mice*_ *=* 5/group; *n*_*cells*_ *=* 17–20/group. **(C)** T2D cardiomyocytes’ viability was significantly less than in control cardiomyocytes (*p* < 0.001). Naringin treatment increased T2D cardiomyocytes’ viability compared to naringin-untreated cardiomyocytes (*p* < 0.001). Cardiomyocyte viability was not significantly different in the naringin group compared to the naringin-untreated control group (*p* = 0.91 compared to untreated). Data for cell viability was obtained from *n*_*mice*_ *=* 5*/*group and *n*_*cells*_ *=* 19–30/group. All values were normalized to control untreated cardiomyocytes and expressed as means ± S.D. Statistical analysis was performed as described before, ns *p* > 0.05, ***p < 0.001.

The calpains represent a family of Ca^2+^-dependent proteases implicated in the pathophysiology of several inflammatory disorders of the cardiovascular system, including diabetic cardiomyopathy (Wan et al., 2015). Calpain activity was elevated by 228% in untreated T2D cardiomyocytes compared to control cardiomyocytes (*p* < 0.001) (Figure 5A). Naringin treatment (60 mg/kg/d) significantly reduced calpain activity by 35% in T2D cardiomyocytes (*p* < 0.001 compared to untreated diabetic cardiomyocytes) (Figure 5A) but did not change calpain activity in control cells (*p* = 0.99) (Figure 5A).

### Naringin Attenuated Cardiac Injury and Increased Cell Viability in T2D Cardiomyocytes

Chronic hyperglycemia provokes an increased cardiac injury and decreased cardiomyocyte viability (Tan YY. et al., 2020). LDH leak from T2D cardiomyocytes was significantly increased (194%) compared to control cardiomyocytes (*p* < 0.001) (Figure 5B), and naringin treatment (60 mg/kg/d) significantly reduced LDH leak in T2D cardiomyocytes by 53% (*p* < 0.001) compared to untreated T2D cardiomyocytes but did not affect the LDH leak in control cardiomyocytes (*p* = 0.81) (Figure 5B).

T2D cardiomyocyte viability, as assessed by MTT, was 41% lower than in control cardiomyocytes (*p* < 0.001) (Figure 5C). Naringin treatment (60 mg/kg/d) increased T2D cardiomyocytes’ viability by 37% compared to naringin-untreated T2D cardiomyocytes (*p* < 0.001) (Figure 5C). Cardiomyocyte viability in controls was not significantly different in the naringin group compared to the naringin-untreated group (*p* = 0.91) (Figure 5C).

### Naringin Enhanced the Expression of the K_ATP_ Channel

**FIGURE 6:**
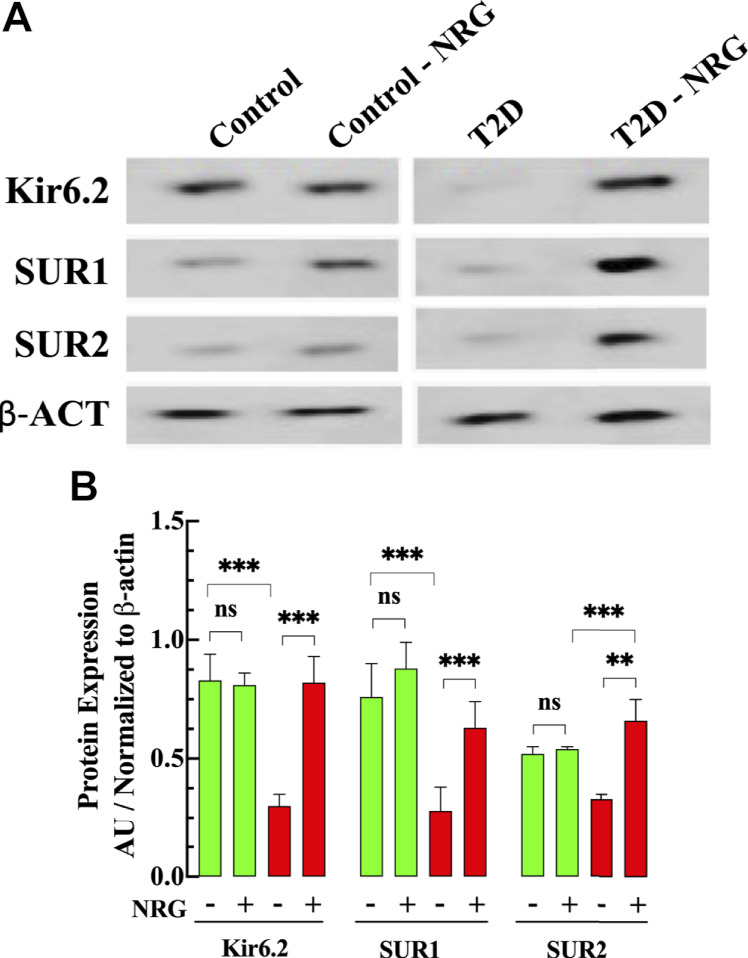
Naringin increases KATP protein expression in diabetic cardiac tissue. **(A)** Representative Western blot bands of Kir6.2, SUR1, and SUR2 subunits expressions in ventricular tissues from naringin treated and untreated control and T2D mice. **(B)** Densitometric analysis of the expression of Kir6.2, SUR1, and SUR2 subunits in T2D show a significant downregulation compared to control tissue (64, 63, and 37%, respectively). Naringin treatment significantly increased the expression of Kir6.2 (173%), SUR1 (130%), and SUR2 (100%) subunits compared with untreated T2D ventricular homogenate. The SUR2 expression in naringin treatment-T2D was greater than control by 26% and control naringin-treated. by 22% (*p* < 0.001). No effect on K_ATP_ channels subunits in control ventricular tissues was observed. Data were normalized to housekeeping protein ß-actin and expressed as means ± S.D. *n*_*mice*_ = 3, *n*_*westerns*_ = 3–4. Statistical analysis was done using paired Student’s independent *t*-test, ns *p* > 0.05, **p < 0.01, ***p < 0.001.

Figure 6 shows a representative Western blot from ventricular homogenates obtained from untreated and naringin-treated control and T2D mice examining the protein expression level of K_ATP_ channel subunits. Our study shows that Kir6.2, SUR1, and SUR2 subunits expression were downregulated in T2D myocardium by 64, 63, and 37%, respectively, compared to control (*p* < 0.001) (Figures 6A,B). Naringin treatment (60 mg/kg/d) induced a significant upregulation of the subunits Kir6.2 (173%), SUR1 (130%), and SUR2 (100%) compared with untreated T2D ventricular homogenates. Interestingly, the expression of SUR2 subunit in naringin-treated T2D was significantly greater than control and control naringin-treated, respectively (26 and 22% respectively, *p* < 0.001). No significant effect was observed on the subunit’s expression levels in control naringin-treated ventricular tissue compared to untreated (*p* = 0.67, *p* = 0.06, and *p* = 0.44, respectively) (Figures 6A,B).

### Glibenclamide Aggravated [Ca^2+^]_d_ Dysfunction and Blocked the Naringin Cardioprotective Effect

**FIGURE 7:**
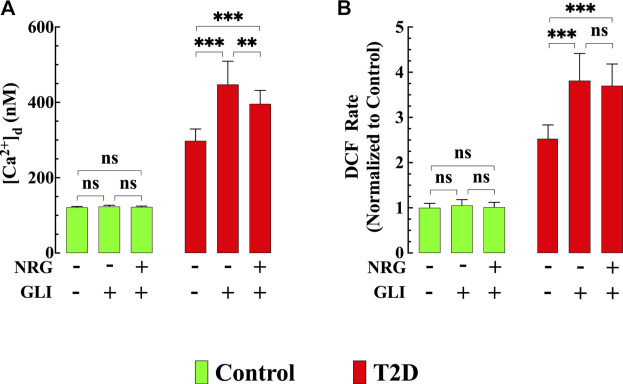
Glibenclamide worsened diastolic [Ca^2+^] and ROS dysfunction and blocked naringin cardioprotection in T2D cardiomyocytes*.* In T2D cardiomyocytes, glibenclamide induced a further significant elevation of [Ca^2+^]_d_ (*p* < 0.001) and ROS generation (*p* < 0.001) compared to untreated T2D cardiomyocytes **(A,B)** and blocked the cardioprotective effects of naringin on [Ca^2+^]_d_, and ROS generation observed in T2D cardiomyocytes **(A,B)**. In control cardiomyocytes glibenclamide did not modify [Ca^2+^]_d_ (*p* = 0.06) and DCF rate (*p* = 0.99). Experimental data were obtained from *n*_*mice*_ *=* 5 mice/group*; n*_*cells*_ = 15–21/group. Values are expressed as means ± S.D. Statistical analysis was performed as described before, ns *p* > 0.05, **p < 0.01, ***p < 0.001.

To test the role of K_ATP_ channels in naringin cardioprotective effect control and T2D mice were treated with glibenclamide, a non-specific K_ATP_ channel blocker (Findlay, 1992). Glibenclamide (10 μg/kg, i. p./d) induced an increase in [Ca^2+^]_d_ in T2D cardiomyocytes from 298 ± 31 nM to 448 ± 62 nM (*p* < 0.001 compared to untreated T2D cardiomyocytes) (Figure 7A) and significantly enhanced ROS production by 47% in T2D cardiomyocytes (*p* < 0.001 compared to untreated T2D cardiomyocytes) (Figure 7B). However, it did not modify either [Ca^2+^]_d_ or ROS production in control cardiomyocytes (Figures 7A,B). Furthermore, glibenclamide prevented the previously observed beneficial effects of naringin on [Ca^2+^]_d_ and ROS production in T2D cardiomyocytes (for comparison, see Figures 1F, 3B).

### Nicorandil Effect on [Ca^2+^]_d_ in T2D Cardiomyocytes

**FIGURE 8:**
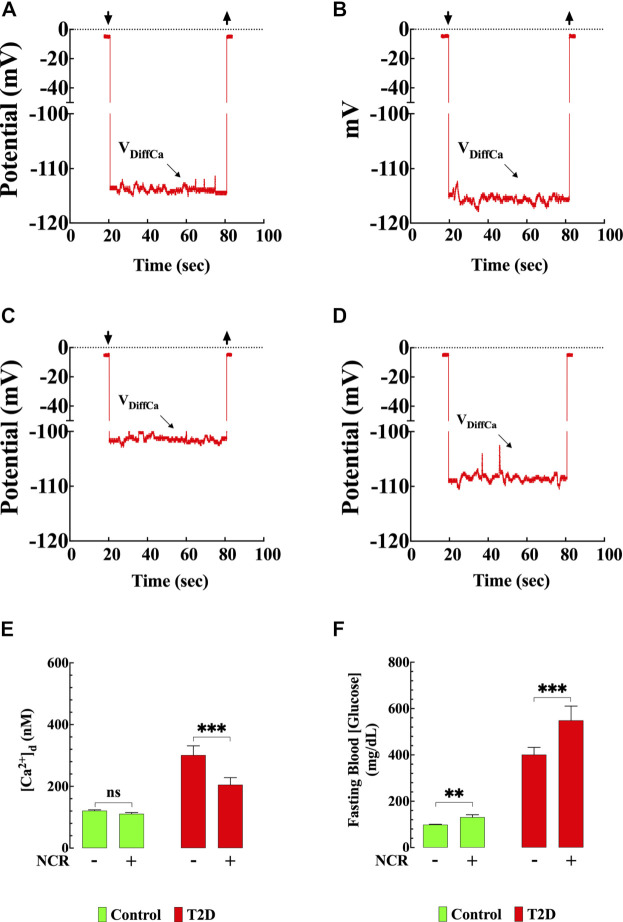
Nicorandil resembled the naringin effects on [Ca^2+^]_d_. Representative recording of [Ca^2+^]_d_ from mouse ventricular cardiomyocytes using double-barreled ion-selective microelectrodes. **(A)** Control with no therapeutic intervention, [Ca^2+^]_d_ = 121 nM. The top left and right arrows represent the Ca^2+^ microelectrode impalement and withdrawing. **(B)** Control treated with nicorandil, [Ca^2+^]_d_ = 117 nM. **(C)** T2D with no therapeutic intervention, [Ca^2+^]_d_ = 315 nM. **(D)** T2D treated with nicorandil, [Ca^2+^]_d_ = 205 nM. **(E)** Summary of nicorandil effect on [Ca^2+^]_d_ in cardiomyocytes. **(F)** Fasting blood glucose values from nicorandil-treated and untreated-control and T2D mice. For the [Ca^2+^]_d_ measurements, *n*_*mice*_ *=* 5*/*group and *n*_*cells*_ *=* 13–18/group. For the fasting glucose determination, *n*_*mice*_ *=* 5*/*group and *n*_*measurements*_ *=* 10/group. Values are expressed as means ± S.D. Statistical analysis was performed as described before, ns *p* > 0.05, **p < 0.01, ***p < 0.001.

To further explore the involvement of the K_ATP_ channel on the naringin cardioprotection, we tested whether nicorandil, a K_ATP_ channel opener and nitric oxide (NO) donor (Uchida et al., 1978; Holzmann, 1983; Ishii et al., 2005; Li et al., 2015) mimics the naringin effect on [Ca^2+^]_d_ in T2D cardiomyocyte. Figures 8A–D show a typical nicorandil effect on [Ca^2+^]_d_ in control and T2D cardiomyocytes. Nicorandil (10 mg/kg/d) in T2D cardiomyocytes reduced [Ca^2+^]_d_ from 301 ± 30 nM to 205 ± 23 nM (32%) (*p* < 0.001) (Figure 8E), and in control, reduced non significantly [Ca^2+^]_d_ from 121 ± 3 nM to 111 ± 4 nM (8%) (*p* = 0.46). The effect of nicorandil on [Ca^2+^]_d_ appears to be mediated mainly through its action on the K_ATP_ channel since incubation of T2D cardiomyocytes in glibenclamide (10 µM) inhibited the nicorandil (300 µM) effects on [Ca^2+^]_d_ in T2D (Supplementary Figure S1). In addition, nicorandil modified glucose homeostasis in control and worsened hyperglycemia in T2D mice. In T2D mice fasting blood glucose level was elevated from 401 ± 31 mg/dl to 549 ± 62 mg/dl after nicorandil (*p* = 0.001), while in control was raised from 99 ± 2 mg/dl to 131 ± 15 mg/dl (*p <* 0.01) upon completion of nicorandil treatment (Figure 8F).

## Discussion

The most significant findings are as follows:

1. Cardiomyocytes and myocardium from T2D mice show i) [Ca^2+^]_d_ overload; ii) Reduced glucose uptake; iii) Elevated ROS generation; iv) Higher myocardial levels of TNF-α and IL-6 and expression of NF-κB; v) Increased calpain activity, cell injury, and reduced cell viability; vi) Reduced protein expression of K_ATP_ channel Kir6.2, SUR1, and SUR2 subunits.
2. Naringin exerts cardioprotection by i) Reducing the [Ca^2+^]_d_ overload; ii) Improving the cardiomyocyte glucose transport; iii) Decreasing ROS production; iv) Reducing myocardial levels of TNF-α and IL-6 and expression of NF-κB. These effects result in an attenuation of cardiomyocyte calpain activity, injury, and increased cell viability. In addition, naringin restores protein expression of K_ATP_ channel subunits Kir6.2, SUR1, and SUR2.
3. Administration of the K_ATP_ channel blocker glibenclamide aggravated [Ca^2+^]_d_ and ROS overload in T2D cardiomyocytes and blocked the ability of naringin to reduce [Ca^2+^]_d_ and ROS excess. Furthermore, the administration of nicorandil, a K_ATP_ channel agonist, mimicked the effect of naringin in T2D on [Ca^2+^]_d_ but induced a further elevation of glucose in T2D and hyperglycemia in control mice.

### Diabetic Cardiomyopathy

DCM is a particular form of heart disorder observed in diabetic patients due to an impairment of diastolic cardiac contractile function and occurs independently of other cardiac risk factors. The probability of developing cardiac dysfunction and heart failure is much higher in T2D patients than in non-diabetic subjects (Radovits et al., 2009; Bouthoorn et al., 2018). The pathophysiological mechanisms of DCM remain poorly understood, and multiple factors such as hyperglycemia, intracellular Ca^2+^ dysfunction, oxidative stress, endoplasmic reticulum stress, inflammation, and mitochondrial alterations have been associated with this pathology (Tan Y. et al., 2020). Pharmacological treatment has substantially improved blood glucose levels in T2D patients’ however, their predisposition to developing cardiovascular disease is much higher than non-diabetic subjects, independent of other comorbidities (Jia et al., 2018).

### Naringin Improved Cardiomyocytes’ Basal Glucose Transport and Lowered Blood Glucose

Diabetes results from a decreased glucose uptake into insulin-sensitive tissues (Defronzo, 2009). In the current study, T2D cardiomyocytes had a significantly reduced glucose uptake compared to control, and naringin improved the depressed T2D cardiomyocyte’s glucose transport. Furthermore, naringin significantly lowered the blood glucose level in T2D mice. Although the downstream pathways by which impaired glucose transport contributes to diabetes are still poorly understood, it is widely accepted that a decrease in GLUT4 expression or deficient translocation plays a critical role (Stenbit et al., 1997). Thus, overexpression of GLUT4 in the db/db mice regularizes the anomalous glucose blood levels and cardiac contractile dysfunction (Belke et al., 2000). Naringin improved the depressed T2D cardiomyocyte’s glucose transport, which could be related to an increase in GLUT4 expression and/or its antioxidant effects on GLUT4 translocation (Dhanya et al., 2015).

### Naringin Effect on [Ca^2+^]_d_ in T2D Cardiomyocytes

We found that cardiomyocytes from T2D mice showed an impaired diastolic Ca^2+^ regulation compared to controls. Intracellular Ca^2+^ dysregulation has been implicated as an important cause of DCM (Levy et al., 1994; Sheikh et al., 2012). In healthy cardiomyocytes, diastolic [Ca^2+^] is in the range of 100–120 nM (Marban et al., 1980; Mijares et al., 2014). It is maintained through the interplay of Ca^2+^ transport mechanisms and ion channels, including sarcolemma and intracellular organelles, Ca^2+^ influx-efflux, and ryanodine receptor-mediated Ca^2+^ leak (Bers and Ginsburg, 2007; Bers, 2014; Freichel et al., 2017; Smith and Eisner, 2019). Although the specific mechanism underlying the diastolic Ca^2+^ alterations in T2D cardiomyocytes have not been established yet, cardiac dysfunction in diabetes has been associated with detrimental expression of the sarcoplasmic reticulum Ca^2+^ pump SERCA2 (Belke et al., 2004; Suarez et al., 2008) and an aberrant ryanodine receptor Ca^2+^ leak (Santulli et al., 2015). This study revealed that naringin treatment significantly reduced [Ca^2+^]_d_ overload in T2D cardiomyocytes, which had never been investigated previously. This protective effect could be attributed to a combination of its antioxidant and anti-inflammatory effects as well as its ability to enhance K_ATP_ channel expression, as we found in the present study. However, we discerned that the effect of naringin on [Ca^2+^]_d_ is not linked to its potential insulinotropic effect (Hameed, 2018) because, in isolated cardiomyocytes from T2D mice (devoid of any insulin action), we observed a dose-dependent reduction of [Ca^2+^]_d_ when incubated with naringin (100–300 µM) (Supplementary Figure S2).

### Oxidative Stress, Inflammation, Cardiac Injury, and Cell Viability

A significant finding of the present study is the increase in ROS generation in cardiomyocytes isolated from T2D mice. It is widely accepted that increased ROS plays a vital role in the pathogenesis of DCM (Evans et al., 2003). Oxidative stress appears to generate insulin resistance by altering intracellular signaling, including increased ryanodine receptor phosphorylation and downregulation of sarco-endoplasmic reticulum Ca^2+^-ATPase transcription (Evans et al., 2003). The present study revealed that naringin induced a marked reduction of ROS generation, an important hallmark in T2D. These findings concur with previous reports about the antioxidant effects of naringin in diverse tissues (Dhanya et al., 2015), which appear to be mediated by increasing activities of catalase and superoxide dismutase (Kwatra et al., 2016), suppressing proinflammatory cytokine production (Marfella et al., 2003) and thus preventing mitochondrial ROS generation, rather than enhancing cellular ROS removal systems (Facundo et al., 2006). Furthermore, recent studies have underscored the notion that Ca^2+^ and ROS signaling systems are intimately integrated, such that Ca^2+^-dependent regulation of components of ROS homeostasis might influence intracellular redox balance and vice versa (Brookes et al., 2004). Thus, the reduction of ROS generation by naringin may be a consequence of lowering [Ca^2+^]_d_ and/or the activation of K_ATP_.

Proinflammatory cytokines, like TNF-α and IL-6, and the transcriptional factor NF-κB are involved in the pathogenesis of DCM (Lorenzo et al., 2011; Palomer et al., 2013; Grubic Rotkvic et al., 2021). Our results revealed that TNF-α and IL-6, and NF-κB were elevated in the T2D myocardium, which confirms the involvement of inflammation in the pathogenesis of diabetes-induced cardiac injury (Grubic Rotkvic et al., 2021). Activation NF-κB is one of the key transcription factors involved in the expression of proinflammatory cytokines and hypertrophy-related genes (Yang et al., 2009). Its activation in the diabetic heart causes the release of interleukins and TNF-α and amplifies oxidative stress resulting in endothelial dysfunction in T2D (Yang et al., 2009). Naringin demonstrated its cardioprotective nature by reducing the content of proinflammatory cytokines and the expression of transcriptional factor NF-κB, actively involved in developing T2D-induced cardiomyopathy (Lorenzo et al., 2011).

In addition, naringin’s effects were accompanied by a decrease in the elevated calpain activity in T2D treated cardiomyocytes. Calpains are a family of Ca^2+^-dependent serine proteases, which play a vital role in physiological and pathological processes, including diabetes mellitus and ischemia (Wan et al., 2015). Calpains activation by Ca^2+^ causes proteolysis-induced cardiomyocyte injury and death (Trumbeckaite et al., 2003). The elevated calpain activity agrees with previous studies, which showed calpain activation in cultured endothelial cells and vascular tissues from T1D and T2D subjects (Wang et al., 2008; Chen B. et al., 2014). The effect of naringin on calpain activity could be mediated by reducing [Ca^2+^]_d_ elicited by this flavonoid.

T2D cardiomyocytes showed an elevated index of cell damage evidenced by an elevated LDH leak and lower cell viability (MTT assay) compared to control cardiomyocytes. Naringin significantly lowered LDH leak (cardiomyocyte injury) and increase cell viability compared to untreated T2D cardiomyocytes. It is plausible to suggest that both of these naringin effects could be the result of the reduction of [Ca^2+^]_d_ since intracellular Ca^2+^ dyshomeostasis can activate pro-apoptotic or necrosis signaling pathways, which will reduce cell viability, as has been shown in diabetic myocardium (Frustaci et al., 2000; Cai et al., 2002).

### Effects of Naringin on the Expression of Cardiac K_ATP_ Channels

Our study shows a reduction in the expression of Kir6.2, SUR1, and SUR2 subunits of K_ATP_ channels in T2D cardiac cells. Our data partly concur with a previous report by Fancher (Fancher et al., 2013), who studied the expression of Kir6.1 and SUR1 and found a reduction of both subunits’ expression in diabetic cardiomyocytes. K_ATP_ channels play a key role in regulating cell functions under physiologic and pathological conditions (Noma, 1983; Inagaki et al., 1995). Structurally K_ATP_ channels are composed of two types of subunits, the inwardly rectifying K^+^ channel (Kir6.1, Kir6.2) and the sulphonylurea subunits (SUR1 and SUR2) (Inagaki et al., 1995; Shyng and Nichols, 1997). Interestingly, our study shows that treatment with naringin provoked the upregulation of Kir6.2, SUR1, and SUR2 subunits of the K_ATP_ channels in T2D cardiomyocytes compared with untreated T2D ventricular cells. These findings suggest that the downregulation of cardiac K_ATP_ may play an essential role in the pathophysiology of diabetic cardiomyopathy.

### Glibenclamide Blocks the Effect of Naringin on [Ca^2+^]_d_ in T2D Cardiomyocytes

Our results revealed that K_ATP_ channel blockade with glibenclamide worsened the [Ca^2+^]_d_ dysfunction observed in T2D cardiomyocytes and abolished the above-mentioned cardioprotective effects of naringin on [Ca^2+^]_d_. Based on the present results, it appears that naringin is also acting on a Ca^2+^ pathway non-glibenclamide-sensitive since it could reduce [Ca^2+^]_d_ in T2D cardiomyocytes by (12%) in the presence of glibenclamide. One possibility is that naringin, through its antioxidant effect, also inhibits the sarcolemmal cation (Ca^2+^ and Na^+^) influx activated by oxidative stress as observed in erythrocyte (Brand et al., 2003; Shaik et al., 2012).

This data support the concern about the use of sulfonylurea hypoglycemic agents (first generation) in diabetic patients due to an increased occurrence of adverse cardiovascular outcomes (Pantalone et al., 2009; Tzoulaki et al., 2009; Aronson and Edelman, 2014; Gilbert and Krum, 2015), and the higher hospital mortality after angioplasty myocardial infarction (Garratt et al., 1999; Bell and Goncalves, 2019). However, other studies have suggested that it’s use in patients with T2D is not associated with increased cardiovascular risk and all-cause mortality (Rados et al., 2016). Therefore, the safety of sulfonylurea in diabetic patients remains controversial and routine utilization as second-line agents may be acceptable in the short term, in line with the American Diabetes Association Standards of Care (American Diabetes, 2020).

### Nicorandil Mimics the Effects of Naringin on [Ca^2+^]_d_ in T2D

Nicorandil is a cardioprotective agent with two postulated mechanisms of action: an increased K^+^ conductance of the cardiomyocytes by activation K_ATP_ channel and a NO donor, increasing cyclic guanosine monophosphate concentration in the cardiac cell (Uchida et al., 1978; Holzmann, 1983; Tsuchida et al., 2002; Mccully and Levitsky, 2003). Our data show that nicorandil mimics the effects of naringin lowering [Ca^2+^]_d_ in T2D cardiomyocytes, a pharmacological action that appears to be mediated mainly by K_ATP_ channels since glibenclamide pretreatment inhibited mostly of its effect on [Ca^2+^]_d_ in T2D cardiomyocytes (Supplementary Figure S2). The remained effect of nicorandil on [Ca^2+^]_d_ in the presence of glibenclamide (26%) could be attributed to its NO donor effect (Taira, 1989). Interestingly, in contrast to naringin, nicorandil further elevated blood glucose levels in T2D. These findings allow us to suggest that the effects of naringin and nicorandil on [Ca^2+^]_d_ appear to be mediated mainly by its action of K_ATP_ channels (Mccully and Levitsky, 2003) and not by its NO donor effect.

### Study Limitations

Despite the originality of our study, some limitations should be acknowledged. The cardioprotective effects of naringin were studied in young male T2D mice (12 weeks) and not in females or in aged mice (24 months), which may have more advanced cardiomyopathy. The effect of naringin on cardiomyocyte’s glucose uptake was established only on basal conditions and not upon insulin stimulation. The cardioprotective action of naringin was explored at doses between 20 and 60 mg/kg/d bodyweight but not at higher doses (>60 mg/kg/d). The short-term treatment (4 weeks) did not allow us to establish whether a more extended treatment may provoke greater cardioprotection in T2D cardiac cells. Finally, the involvement of sarcolemmal K_ATP_ or mitochondrial K_ATP_ channels on the naringin cytoprotection was not discerned, and its effect on T2D cardiomyocyte’s contractile response was not studied.

## Concluding Remarks

The present study demonstrated that the citrus fruit flavonoid naringin protects against cardiomyopathy in T2D mice. The mechanism by which naringin exerts its cardioprotective effect appears to be multifaceted. The results of this study relate its cardioprotective effects to the reduction of intracellular Ca^2+^ overload, limiting the increase in ROS generation, reducing calpain activity, TNF-α and IL-6 level, decreasing NF-κB expression, and upregulating K_ATP_ channels. These results suggest that naringin may be a novel therapeutic candidate for protection against diabetes-induced myocardial dysfunction.

## Data Availability

The raw data supporting the conclusions of this article will be made available by the authors, without undue reservation.

## Supplementary Material

Supplemental Figure S1 Glibenclamide partially inhibited the effect of nicorandil on [Ca^2+^]_d_ in T2D cardiomyocytes. To discriminate the mechanisms of action of nicorandil due to K_ATP_ channel-opening properties from those due to its NO-donor properties [Ca^2+^]_d_ was measured in control and T2D isolated cardiomyocytes before and after incubation with either: **(A)** the K_ATP_ channel opener nicorandil (300 µM); (**B)** the K_ATP_ channel antagonist glibenclamide (10 µM); **(C)** glibenclamide (10 µM) for 2 h, followed by nicorandil (300 µM). Nicorandil reduced significantly [Ca^2+^]_d_ in T2D but not in control cardiomyocytes. Glibenclamide produced a further elevation of [Ca^2+^]_d_ in T2D cardiomyocytes but not in controls cardiomyocytes; glibenclamide, followed by nicorandil, significantly reduced the effect of nicorandil on [Ca^2+^]_d_. *n*_*mice*_ *=* 3 controls and 3 T2D mice and *n*_*cells*_ *=* 20–22/group. Values are expressed as means ± S.D. Statistical analysis was performed as described before, ns *p* > 0.05, ****p* < 0.001.

Supplemental Figure S2 | Effects of naringin on [Ca^2+^]_d_. Naringin reduced significantly [Ca^2+^]_d_ in T2D cardiomyocytes in a dose significant manner. Control and T2D cardiomyocytes were exposed to naringin (100, 200, or 300 µM) for 2 h, then measured [Ca^2+^]_d_. Naringin provoked a dose-dependent reduction of [Ca^2+^]_d_ in cardiomyocytes isolated from T2D mice with no effect on control cardiomyocytes. *n*_*mice*_ *=* 3 controls and 4 T2D mice and *n*_*cells*_ *=* 16–20/group. Values are expressed as means ± S.D. Statistical analysis was performed as described before, ns *p* > 0.05, **p* < 0.05, ****p* < 0.001.

## Abbreviations

T2D, type 2 diabetes; [Ca^2+^]_d_, diastolic calcium concentration; KATP, ATP-sensitive potassium; ROS, reactive oxygen species; VCaE, Ca^2+^ potential; VDiffCa, differential Ca^2+^-specific potential; TNF-α tumor necrosis factor-α; IL-6, interleukin-6; NF-κB, transcription nuclear factor-κB; pCa, negative logarithm of Ca^2+^ concentration; NRG, naringin; GLI, glibenclamide; NCR, nicorandil; ACT, Actin.
