# Supplementary figures and images for: Effects of Naringin on Cardiomyocytes From a Rodent Model of Type 2 Diabetes
Source: Front Pharmacol. 2021 Aug 23;12:719268. doi: 10.3389/fphar.2021.719268 (PMC8419284; doi:10.3389/fphar.2021.719268)

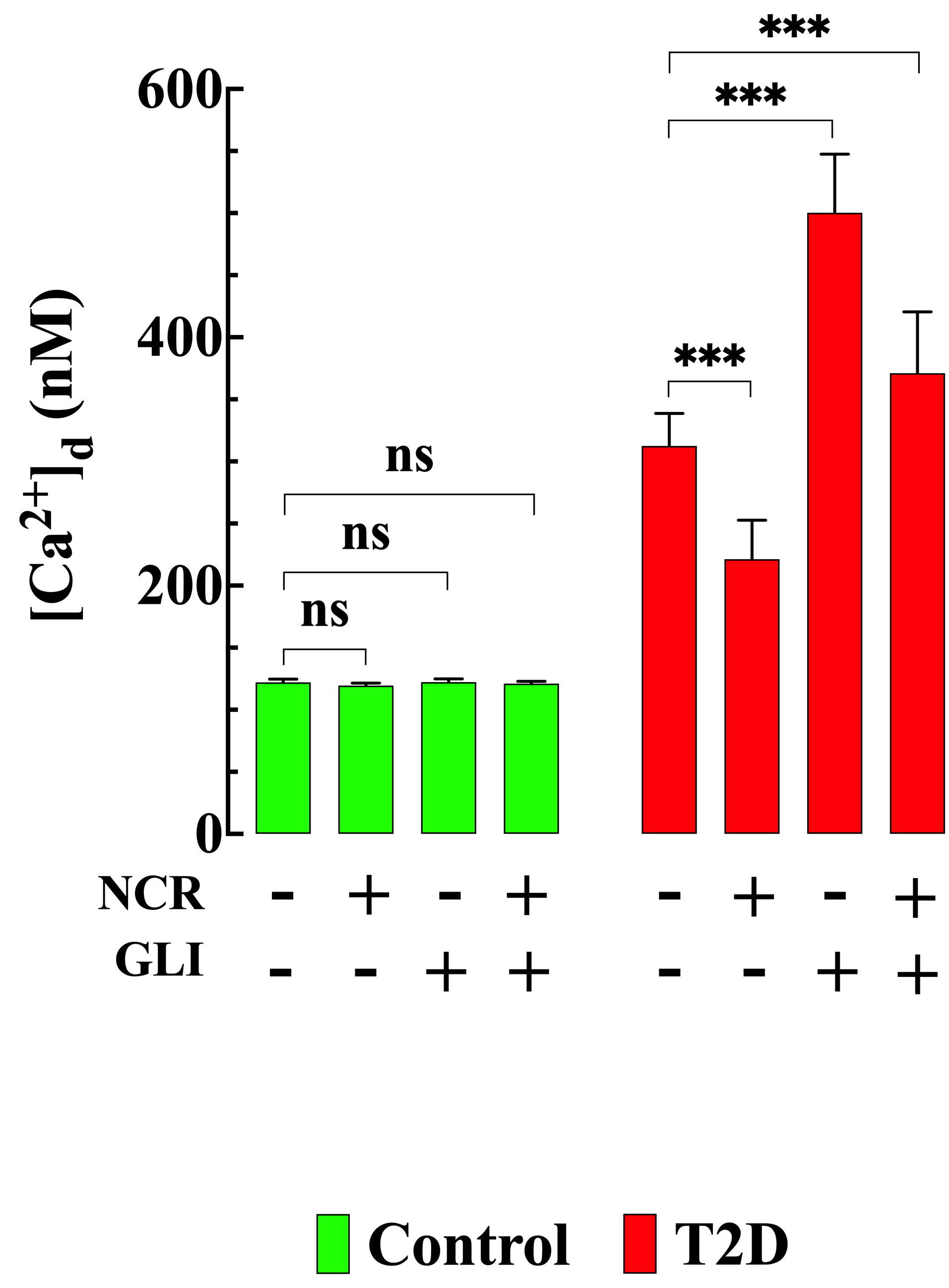

Supplement: Supplementary file 1 [file Image1.tiff]

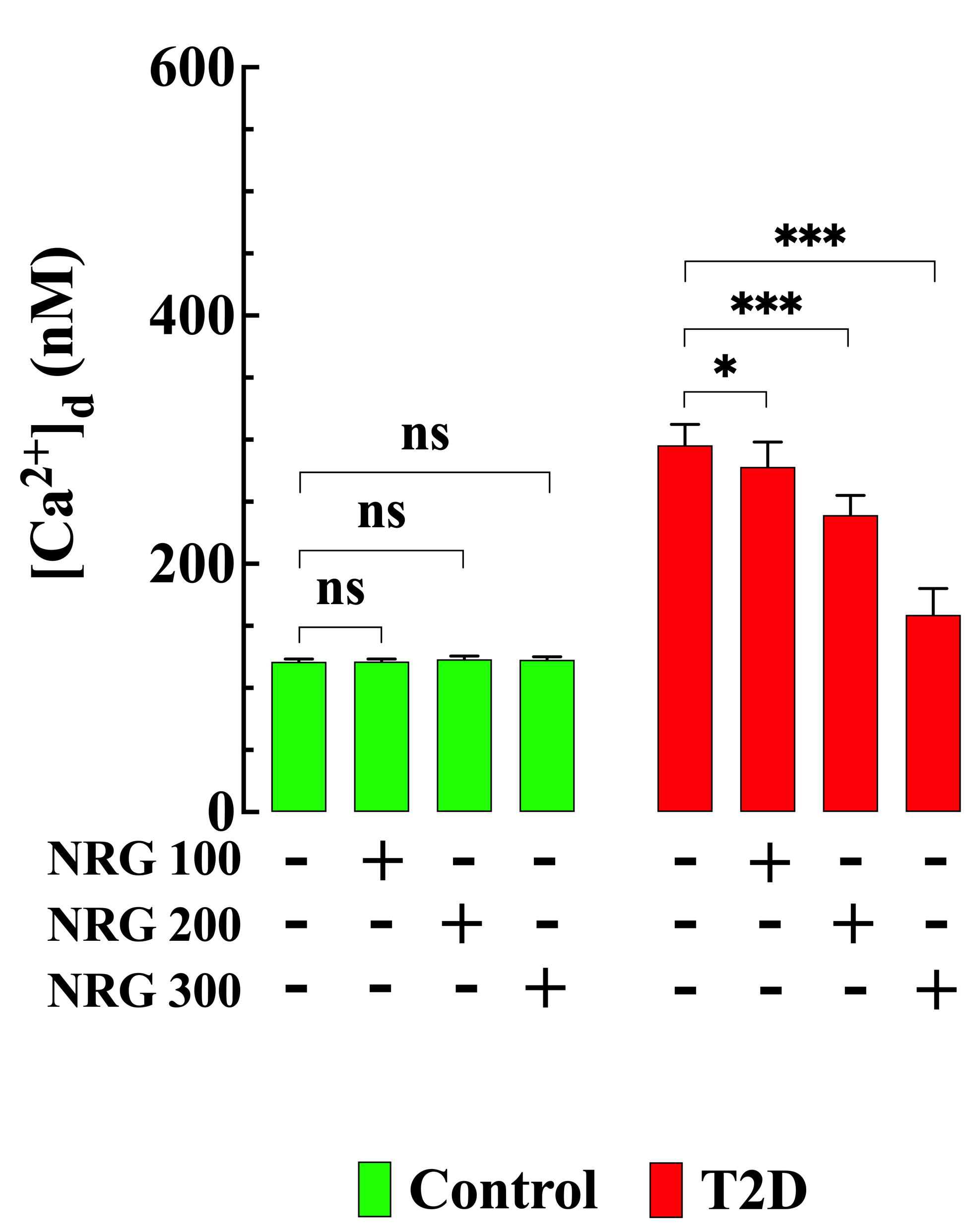

Supplement: Supplementary file 2 [file Image2.tiff]
